# DEPRESCRIBING AMONG OLDER ADULTS WITH CANCER

**DOI:** 10.1093/geroni/igae098.2310

**Published:** 2024-12-31

**Authors:** Martha Coates, Emily Hajjar, Rose Ann DiMaria-Ghalili

**Affiliations:** Drexel University, Philadelphia, Pennsylvania, United States; Thomas Jefferson University, Philadelphia, Pennsylvania, United States; Drexel University, Philadelphia, Pennsylvania, United States

## Abstract

Older adults with cancer experience high rates of polypharmacy (>5 medications daily) and potentially inappropriate medication use which are associated with adverse health outcomes and negatively impact quality of life. Deprescribing, the process of lowering drug exposure or removing medications that could cause harm or no longer provide benefit, can reduce the risk of negative outcomes. There is limited research examining the acceptance toward deprescribing in older adults with cancer, therefore the purpose of this study was to examine deprescribing acceptance among Medicare beneficiaries with cancer. We utilized the National Health and Aging Trends Study Round 6 (2016) dataset and included individuals who answered the Medication Attitudes Module (n=1975). Among the participants, 6.1% (n=121) reported having a history of cancer and over half were experiencing polypharmacy. Older adults with cancer strongly agreed or agreed that they took a large number of medications (63%), would like to reduce their medications (74.4%) and if their doctor suggested it they would stop one or more of their medications (95.8%). Despite these high levels of agreement, participants were also found to disagree they took medications they didn’t need (77%) and highly agreed all of their medications were necessary (93.3%). Older adults with cancer are willing to stop medications they may no longer need if suggested by their healthcare provider, but strongly disagree they are taking medications they do not need. Further research is needed to examine patient perceived facilitators and barriers to deprescribing medications that may no longer be needed in individuals with cancer.

